# *Klebsiella pneumoniae* Epidural Abscess Presenting as Tubercular Spondylodiscitis: A Diagnostic Pitfall in a Diabetic Patient From Nepal

**DOI:** 10.1002/ccr3.73777

**Published:** 2026-10-23

**Authors:** Rohit Rawat, Shitanshu Dhakal, Shankar Poudel

**Affiliations:** ^1^ Patan Academy of Health Sciences Lalitpur Nepal; ^2^ Department of Surgery Tribhuvan University Teaching Hospital, Institute of Medicine, Tribhuvan University Kathmandu Nepal

**Keywords:** diabetes mellitus, *Klebsiella pneumoniae*, spinal epidural abscess, spondylodiscitis, tuberculosis mimicker

## Abstract

*Klebsiella pneumoniae* spinal epidural abscess is rare and can mimic tuberculous spondylodiscitis in endemic regions. We report a diabetic patient with pulmonary tuberculosis presenting with lumbar spondylodiscitis and epidural abscess. CT‐guided biopsy identified *Klebsiella pneumoniae* , with no evidence of tuberculosis. Ceftriaxone led to recovery, highlighting the importance of tissue‐based diagnosis.

## Key Clinical Message

In tuberculosis‐endemic settings, spinal epidural abscess and spondylodiscitis should not be presumed to be tuberculous based on imaging alone, even in patients with concurrent pulmonary tuberculosis. Tissue‐based diagnosis is crucial for identifying alternative pathogens such as *Klebsiella pneumoniae* and guiding appropriate antimicrobial therapy.

## Graphical abstract

MRI showing spinal epidural abscess initially suspected to be tuberculous spondylodiscitis. CT‐guided biopsy identified Klebsiella pneumoniae, highlighting the importance of tissue‐based diagnosis in guiding appropriate treatment.

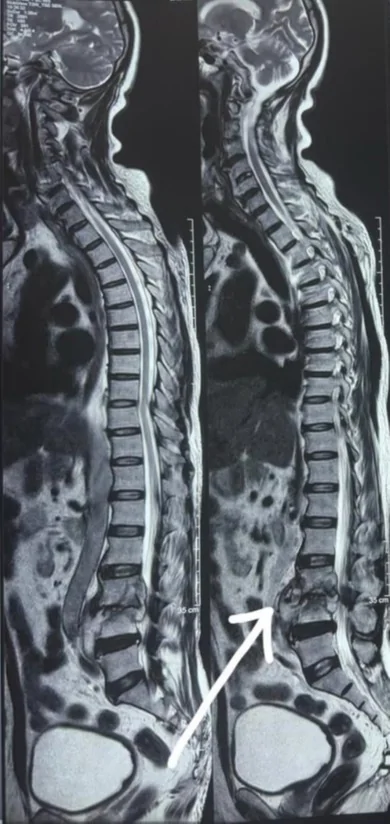

## 1 Introduction

Spinal epidural abscess (SEA) and spondylodiscitis are uncommon but potentially life‐threatening conditions that can result in significant neurological morbidity if not diagnosed and treated promptly [1]. The incidence of SEA has been rising over recent decades, largely attributable to the increasing prevalence of predisposing conditions such as diabetes mellitus, immunosuppression, intravenous drug use, spinal instrumentation procedures, and the improved sensitivity of magnetic resonance imaging (MRI) in detecting spinal infections [2].

Spondylodiscitis refers to infection involving the intervertebral disc and the adjacent vertebral bodies, and may extend into the epidural space forming an abscess, or into the paravertebral musculature forming a psoas collection. The condition typically presents with subacute, progressively worsening axial back pain, elevated inflammatory markers, and characteristic findings on MRI. Diagnostic delays are frequent, with the time from symptom onset to diagnosis often spanning weeks to months [3].

*Staphylococcus aureus* remains the most commonly identified causative organism in SEA, accounting for approximately 60%–66% of cases [4]. Gram‐negative bacteria constitute approximately 16% of SEA cases overall, and among these, *Klebsiella pneumoniae* accounts for only around 1%, making it an exceedingly rare but increasingly recognized pathogen, particularly in diabetic and elderly patients [5].

*Klebsiella pneumoniae* is a member of the Enterobacterales family and is capable of causing a spectrum of community‐acquired and healthcare‐associated infections including pneumonia, urinary tract infections, liver abscesses, and bacteremia [6]. Hematogenous seeding of the spinal column from a distant infectious focus represents one of the primary routes by which Klebsiella reaches the epidural space [4]. Diabetes mellitus is recognized as a significant independent risk factor for both SEA and Klebsiella infections, as impaired cellular immunity and microvascular disease create a permissive environment for invasive bacterial infection [6].

In tuberculosis‐endemic regions such as Nepal, the radiological appearance of spondylodiscitis with epidural and psoas collections poses a particularly challenging diagnostic dilemma, as these findings are characteristic of both tuberculous (Pott's disease) and pyogenic spinal infections. The co‐existence of active pulmonary tuberculosis in such patients further compounds this diagnostic uncertainty and may lead to empirical anti‐tubercular therapy without tissue‐based microbiological confirmation [7].

We present a rare case of *Klebsiella pneumoniae* epidural abscess and spondylodiscitis in a diabetic Nepali patient with concurrent pulmonary tuberculosis, whose radiological presentation mimicked tuberculous spondylodiscitis. This case underscores the critical importance of tissue‐based diagnosis and the need to consider gram‐negative bacterial pathogens, particularly in diabetic patients presenting with spinal infection in tuberculosis‐endemic settings. This case report has been prepared in accordance with the CARE reporting guidelines [8].

## 2 Case Presentation

### 2.1 Case History

A 65‐year‐old male with a background of well‐managed type 2 diabetes mellitus and recently diagnosed pulmonary tuberculosis presented with a five‐month history of progressively worsening low back pain radiating to the right lower limb. The pain was only partially relieved by rest and had been gradually increasing in severity, significantly affecting his daily activities.

### 2.2 Examination

On physical examination, the patient was haemodynamically stable and alert. Spinal tenderness was elicited on palpation at the L3–L4 level. The straight leg raise test exceeded 70 degrees bilaterally. Neurological examination revealed intact motor and sensory function in both lower limbs, with no bowel or bladder dysfunction. Bilateral Babinski reflexes were downgoing, confirming preserved upper motor neuron function. All other systemic examinations were unremarkable.

### 2.3 Differential Diagnosis, Investigations and Treatment

The initial clinical consideration included tuberculous spondylodiscitis given the patient's background of pulmonary tuberculosis and the endemic prevalence in Nepal, alongside other possibilities such as pyogenic spondylodiscitis. Laboratory investigations revealed elevated erythrocyte sedimentation rate (ESR) and C‐reactive protein (CRP), while blood cultures were repeatedly sterile. MRI and CT imaging of the lumbosacral spine demonstrated L3–L4 spondylodiscitis with a significant epidural abscess and associated psoas collection (Figure 1), radiologically suggestive of tuberculous spondylodiscitis.

**FIGURE 1:**
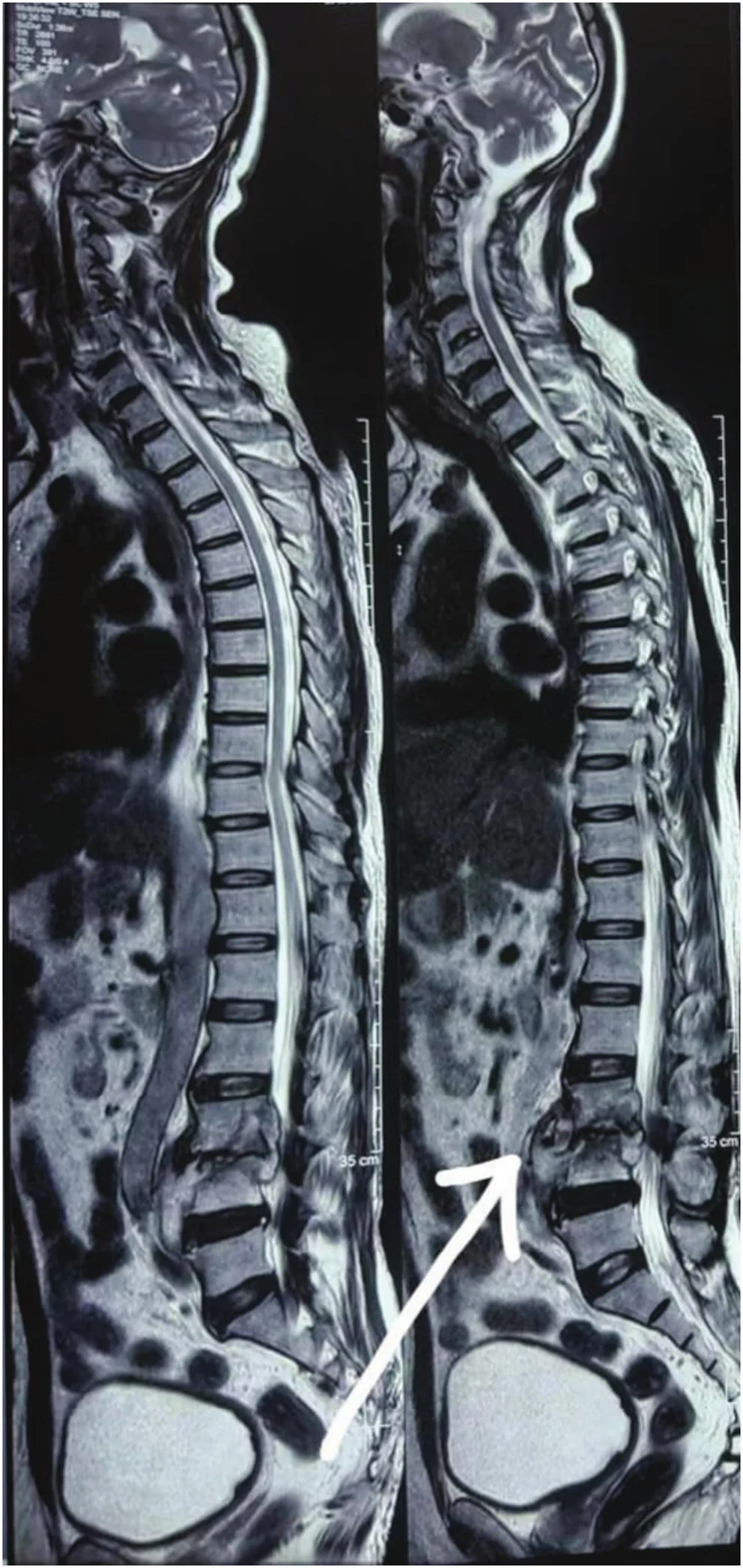
MRI of the lumbosacral spine demonstrating L3–L4 spondylodiscitis with a large epidural abscess and associated psoas collection (arrow), indicative of advanced spinal infection.

However, to establish a definitive diagnosis, a CT‐guided bone biopsy was performed. Microbiological culture of the biopsy specimen grew *Klebsiella pneumoniae* . PCR for *Mycobacterium tuberculosis* was negative, and histopathology showed no granulomatous inflammation, together providing no microbiological or histopathological evidence of spinal tuberculosis. The patient was managed conservatively without surgical intervention. Following multidisciplinary discussion, he received culture‐directed intravenous ceftriaxone for 6 weeks, alongside continued anti‐tubercular therapy for his concurrent pulmonary tuberculosis.

### 2.4 Outcome and Follow‐Up

The patient showed marked clinical improvement with progressive resolution of low back pain, normalization of inflammatory markers, and radiological improvement of the epidural and psoas collections on follow‐up imaging. He remained neurologically intact throughout the treatment course and recovered without complications.

## 3 Discussion

This case highlights the diagnostic challenge posed by *Klebsiella pneumoniae* spinal epidural abscess and spondylodiscitis when presenting in a patient with concurrent pulmonary tuberculosis in a tuberculosis‐endemic region. The radiological appearance of L3–L4 spondylodiscitis with epidural and psoas collections was highly suggestive of Pott's disease, and without tissue‐based confirmation, this patient may have been managed exclusively with anti‐tubercular therapy, delaying appropriate antimicrobial treatment for the underlying bacterial infection.

SEA is a rare but serious condition with an incidence of approximately 5.1 cases per 10,000 hospital admissions. Its clinical presentation is frequently non‐specific; the classic triad of fever, back pain, and neurological deficit is present in fewer than 50% of cases, making early diagnosis particularly challenging [2]. In our patient, the absence of fever and intact neurology at presentation represented an atypical picture, consistent with prior literature documenting variable clinical manifestations across reported cases of Klebsiella SEA [6].

Sterile blood cultures, as observed in our patient, are not uncommon in spinal infections and do not exclude a pyogenic etiology. Hematogenous spread from a distant infectious source accounts for nearly half of all SEA cases, yet blood cultures may fail to isolate the causative organism. This underscores the important role of CT‐guided bone biopsy in establishing microbiological diagnosis, particularly when blood cultures are negative or when the clinical and radiological picture is ambiguous [9].

Among gram‐negative organisms causing SEA and spondylodiscitis, *Klebsiella pneumoniae* is exceedingly rare, accounting for approximately 1% of all SEA cases [5]. A literature review by Nitinai et al. identified only 10 published cases of *Klebsiella pneumoniae* SEA, with diabetes mellitus present in half of those cases, confirming its role as a major predisposing factor [6]. Our patient had type 2 diabetes mellitus, a recognized risk factor for invasive Klebsiella infections.

The lumbar spine is the most common location for SEA, accounting for approximately 48% of cases, followed by the thoracic spine at 31% and the cervical spine at 24% [10]. The L3–L4 involvement in our patient is consistent with this distribution. The concurrent psoas collection, a recognized complication of lumbar spondylodiscitis, further complicated the radiological picture and reinforced the initial clinical suspicion of tuberculosis, given that psoas abscess is a classical feature of Pott's disease [7].

The diagnostic pitfall in our case was compounded by the patient's established diagnosis of pulmonary tuberculosis and the endemicity of the disease in Nepal. In tuberculosis‐endemic regions, pyogenic spondylodiscitis, particularly that caused by gram‐negative organisms, is frequently misdiagnosed as tuberculous spondylodiscitis on the basis of imaging alone [7]. Tissue‐based diagnosis through percutaneous biopsy with microbiological culture, PCR for *Mycobacterium tuberculosis* , and histopathological examination for granulomas should be pursued when clinically feasible, rather than relying solely on empirical anti‐tubercular therapy [9].

Regarding management, our patient was treated conservatively with culture‐directed intravenous ceftriaxone for 6 weeks without surgical intervention. Surgical decompression is generally recommended in patients with neurological deficits, spinal instability, or failure of medical therapy. However, several published cases of Klebsiella SEA have demonstrated successful outcomes with antibiotic therapy alone in neurologically intact patients, supporting antibiotic therapy without immediate surgical intervention as a reasonable approach in carefully selected, neurologically intact patients under close monitoring [11].

Ceftriaxone was selected for this patient based on the isolate's susceptibility profile, consistent with its documented efficacy in Klebsiella spinal infections [12]. In cases involving extended‐spectrum beta‐lactamase (ESBL)‐producing strains of *Klebsiella pneumoniae*, however, ceftriaxone would be inadequate and carbapenem therapy becomes the drug of choice, as demonstrated by Dang and Rajkumar in their report of community‐acquired ESBL‐producing Klebsiella SEA in a diabetic patient [5].

The multidisciplinary approach adopted in this case involving spinal surgery, infectious disease, and close clinical monitoring is consistent with best practice recommendations for the management of spinal infections. Brooks et al. similarly emphasized the importance of multidisciplinary expertise in achieving optimal outcomes in Klebsiella spondylodiscitis, noting that inadequate antimicrobial treatment and delayed surgical intervention can result in catastrophic neurological deterioration [3].

This case also highlights the growing clinical significance of gram‐negative spinal infections globally, with a disproportionate representation of cases from the Asian Pacific region, potentially attributable to the higher prevalence of hypervirulent *Klebsiella pneumoniae* strains in this population [6]. As reported by Panossian et al., heightened clinical awareness of atypical gram‐negative pathogens in SEA, prompt imaging, and culture‐guided antibiotic therapy are critical determinants of favorable outcomes [12]. Taken together, this case reinforces that *Klebsiella pneumoniae* should be considered as a potential causative organism in spinal epidural abscess and spondylodiscitis even when tuberculosis is clinically suspected, and that tissue‐based diagnosis should be pursued when clinically feasible to guide antimicrobial therapy, particularly when imaging findings are ambiguous or blood cultures are negative.

## 4 Conclusion

This case demonstrates that *Klebsiella pneumoniae* should be considered as a causative pathogen in spinal epidural abscess and spondylodiscitis, even in tuberculosis‐endemic regions and in patients with concurrent active tuberculosis. When clinically feasible, tissue‐based diagnosis should be pursued to guide antimicrobial therapy, and gram‐negative organisms should be included in the differential diagnosis of spinal infections in diabetic and elderly patients.

## Author Contributions

**Rohit Rawat:** conceptualization, investigation, writing – original draft, visualization. **Shitanshu Dhakal:** resources, supervision, writing – original draft, investigation. **Shankar Poudel:** writing – original draft, writing – review and editing, methodology, investigation.

## Funding

The authors have nothing to report.

## Ethics Statement

The authors have nothing to report.

## Consent

Written informed consent for publication of this case report and accompanying images was obtained from the patient's legal guardian for publication of this case report and accompanying images. A copy of the written consent is available for review by the Editor‐in‐chief of this journal on request.

## Conflicts of Interest

The authors declare no conflicts of interest.

## Data Availability

The data that support the findings of this study are available on request from the corresponding author. The data are not publicly available due to privacy or ethical restrictions.
